# Supplementary material for: A receptor for the complement regulator factor H increases transmission of trypanosomes to tsetse flies
Source: Nat Commun. 2020 Mar 12;11:1326. doi: 10.1038/s41467-020-15125-y (PMC7067766; doi:10.1038/s41467-020-15125-y)
Supplement: Supplementary file 1 — Supplementary Information [file 41467_2020_15125_MOESM1_ESM.pdf]

Supplementary information:

A receptor for the complement regulator factor H increases transmission of trypanosomes to tsetse flies

Macleod et al.

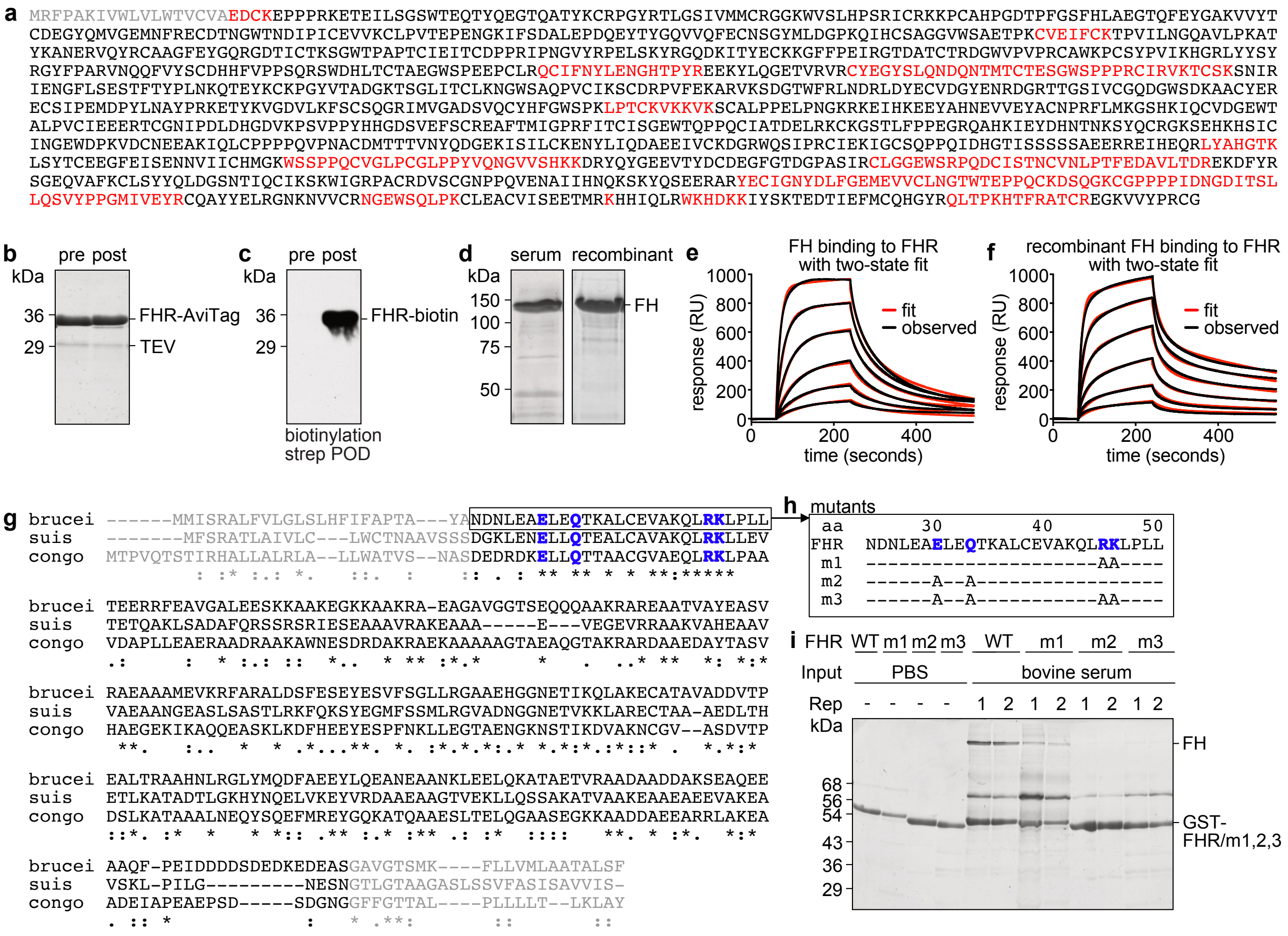

Supplementary Fig. 1 Identification and confirmation of FH as ligand for FHR. a The 145 kDa protein pulled down by FHR was analysed by mass spectrometry and identified as bovine FH (protein score = 3466; significant peptides = 168, threshold = 0.05). Peptides that mapped onto the sequence of bovine FH are shown in black, peptides missing in red, and signal peptide in grey. Sequence coverage was 81.3 %. **b** SDS-PAGE analysis of recombinant FHR used for SPR analysis. Samples are shown before and after biotinylation. **c** Western blot of samples from **b** probed with streptavidin peroxidase. **d** SDS-PAGE analysis of FH purified from bovine serum and recombinant bovine FH. **e** SPR binding data (black) for C-terminally biotinylated FHR (450 RU) bound to a streptavidin chip with two-fold dilutions of FH from bovine serum overlaid with fits (red) to determine the K_D. A two-state binding model was used for data from 1 μM to 31.25 nM producing a K_D of 153 nM. **f** SPR binding data (black) for C-terminally biotinylated FHR (450 RU) bound to a streptavidin chip with two-fold dilutions of recombinant bovine FH (1 μM highest concentration) overlaid with fits (red) to determine the K_D. A two-state binding model was used for data from 1 μM to 31.25 nM producing a K_D of 114 nM. **g** The *T. brucei* FHR sequence was aligned with the closest *T. congolense* (TcIL3000.0.47410) and *T. suis* homologues. Selected conserved residues near the N-terminus were mutated to alanine (blue) in different combinations. Signal peptides are shown in grey. **h** Sequence of first the 27 residues of recombinant FHR and the changes made to produce mutants 1, 2 and 3. **i** SDS-PAGE analysis of pulldowns from bovine serum were performed with GST fusion proteins of wild type FHR and mutants 1, 2 and 3 immobilised on beads. Two replicates for each are shown along with a PBS control. Source data are provided as a Source Data file.

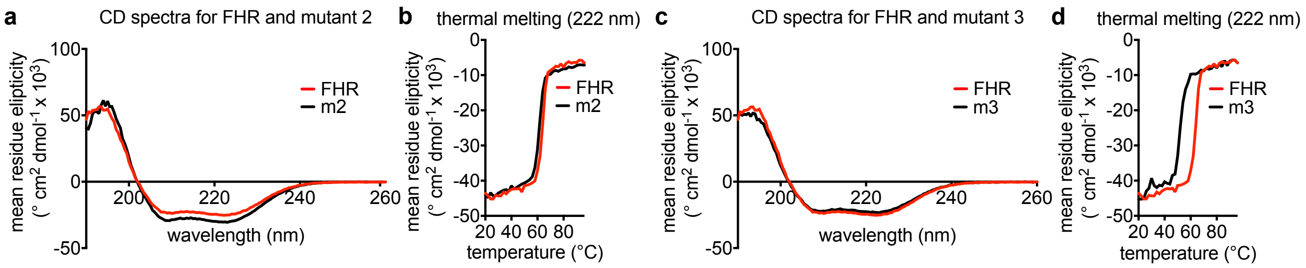

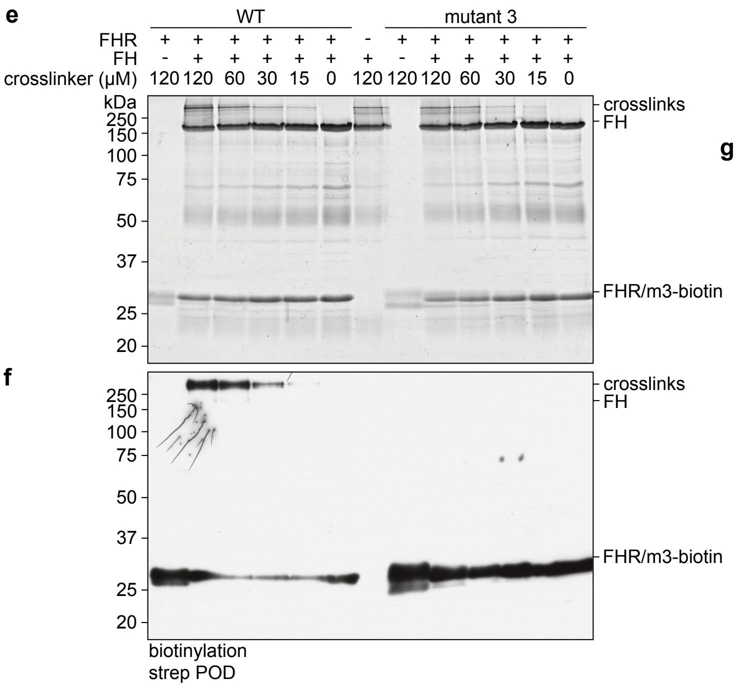

g

FHR res #	FH res #	FH domain	Crosslinked peptides FHR-FH	Seq events
24	283	5	**G**NDNLEAELEQTK – IPNGVYRPELS**K**YR	34
24	1218	20	**G**NDNLEAELEQTK – QLTP**K**HTFR	2
96	444	8	RAEAGAVGGTSEQQQAA**K**R – V**K**TCSK	6
96	448	8	RAEAGAVGGTSEQQQAA**K**R – TCS**K**SNIR	4
120	1195	20	AEAAAMEV**K**R – **K**IYSK	3
120	1218	20	AEAAAMEV**K**R – QLTP**K**HTFR	8

Supplementary Fig. 2 Determination of the FH-FHR binding interface. a Circular dichroism spectra at 20 °C for WT FHR (red) and mutant 2 (black), showing similar profiles with minima 208 and 222 nm, indicative of a high alpha helical content. **b** Thermal melting analysis for WT FHR (red) and mutant 2 (black) by circular dichroism at 220 nm, showing similar profiles and protein folding up to at least 50 °C. Spectra (190 to 250 nm) were recorded starting at 20 °C, followed by 0.5 °C incremental increases to 96 °C. **c** Same as in **a,** but for mutant 3. **d** Same as in **b,** but for mutant 3. **e** SDS-PAGE analysis as in Fig. 1c except with H12disuccimidyl suberate (DSS). **f** Western blot of samples in **e**, and performed as in Fig. 1d except with H12 DSS, probed with streptavidin peroxidase. **g** FHR and FH crosslinked peptides as identified by mass spectrometry. No crosslinked peptides were found in the mutant 3 and FH sample. This data contains all significant crosslinked peptides (e-value < $1x10^{-5}$) found using both D12 and H12 DSS reactions and all crosslinks were shared between the two, except FHR residue 96 to FH residue 444 which was unique to H12 DSS. 'Sequencing events' represents the number of times a crosslinked peptide with a significant score was sequenced. FHR residue 24 is the N-terminal glycine left after TEV protease cleavage and is not part of the native sequence but is numbered accordingly. Red, FHR sequence; Black, FH sequence; Bold letter, crosslinked residue; Black line, crosslink; Res #, residue number. Source data are provided as a Source Data file.

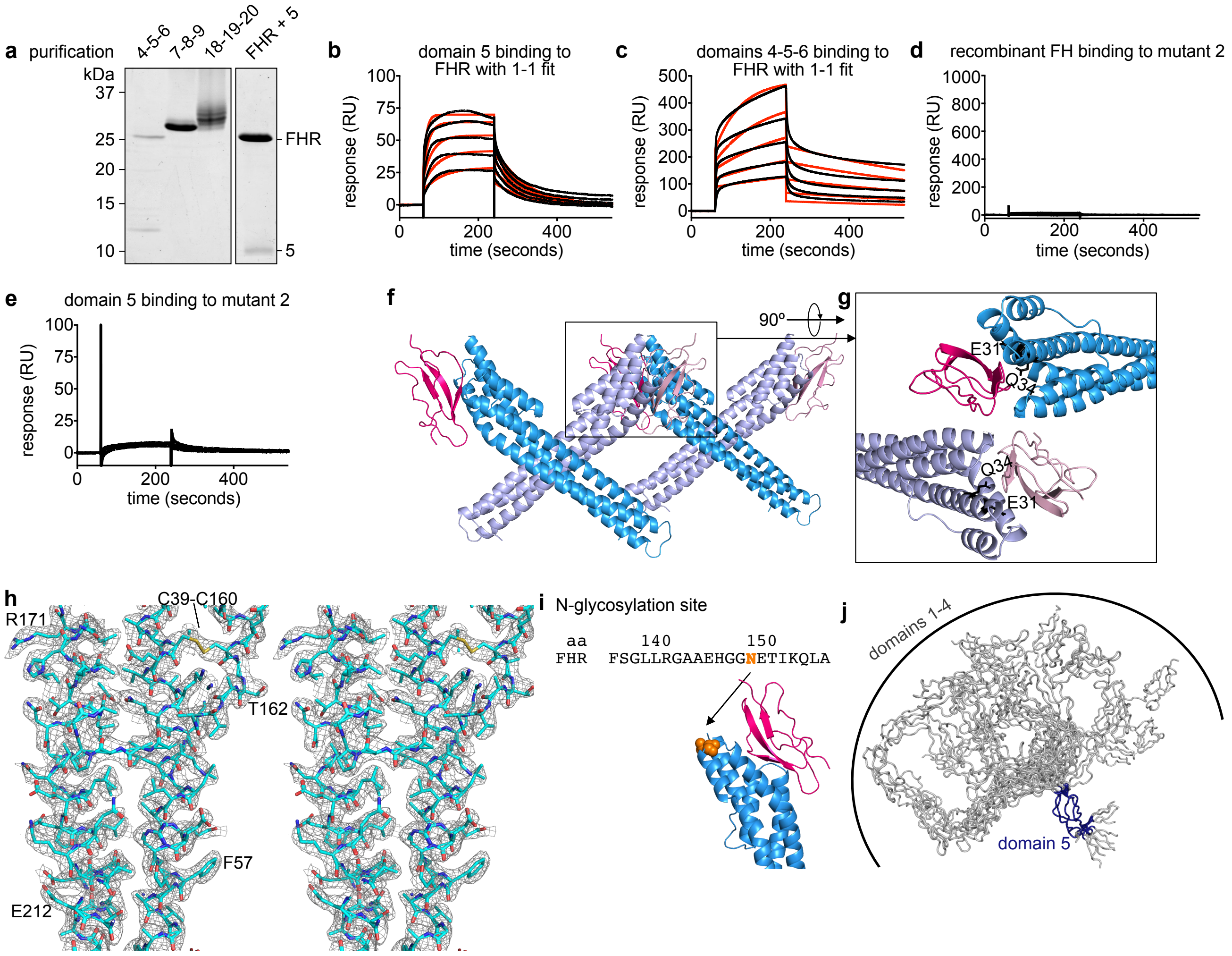

Supplementary Fig. 3 Validation of the interaction between FHR and FH domain 5. a SDS-PAGE of (left) recombinant bovine FH domains 4-5-6, 7-8-9, and 18-19-20 purified from culture supernatant by nickel chromatography, later followed by gel filtration before SPR, and (right) recombinant FHR and bovine FH domain 5, mixed in a 1:1.05 molar ratio, co-purified by gel filtration for crystallography. **b** SPR binding data (black) for C-terminally biotinylated FHR (400 RU) bound to a streptavidin chip with two-fold dilutions of FH domain 5 overlaid with fits (red) to determine the K_D. A 1:1 binding model was used for data from 5 µM to 312.5 nM producing a K_D of 562 nM. **c** Same as **b,** except with FH domains 4-5-6 and data was analysed from 1.5 µM to 93.75 nM, producing a K_D of 167 nM. **d** Same as **b,** except with mutant 2 FHR (400 RU) and two-fold dilutions of recombinant bovine FH with 1 µM as the highest concentration. **e** Same as **d**, except with FH domain 5 and 5 µM as the highest concentration. **f** Two adjacent asymmetric units, each containing two copies of FHR and two copies of domain 5. Complex 1, FHR (blue) and domain 5 (dark pink) and complex 2, FHR (purple) and domain 5 (light pink). Boxed region is zoomed into and rotated in **g**. **g** Rotation of two adjacent asymmetric units showing close proximity of domain 5 from one unit to FHR of the adjacent unit. Residues E31 and Q34 were mutated to alanines (mutant 2) to confirm the identification of the true binding interface. **h** Representative stereo view of electron density map in grey mesh (2Fo−Fc, contour = 1σ) for a cross-section of FHR alpha helices. **i** Location (N150) of the single predicted N-glycosylation site (orange) for FHR. **j** Alignment of solution structures for domains 1-5 of human FH (2QFG) holding domain 5 (dark blue) in place. There is a wide range of conformations for domains 1-4 (grey), demonstrating the flexibility at the domain 4-5 boundary. Source data are provided as a Source Data file.

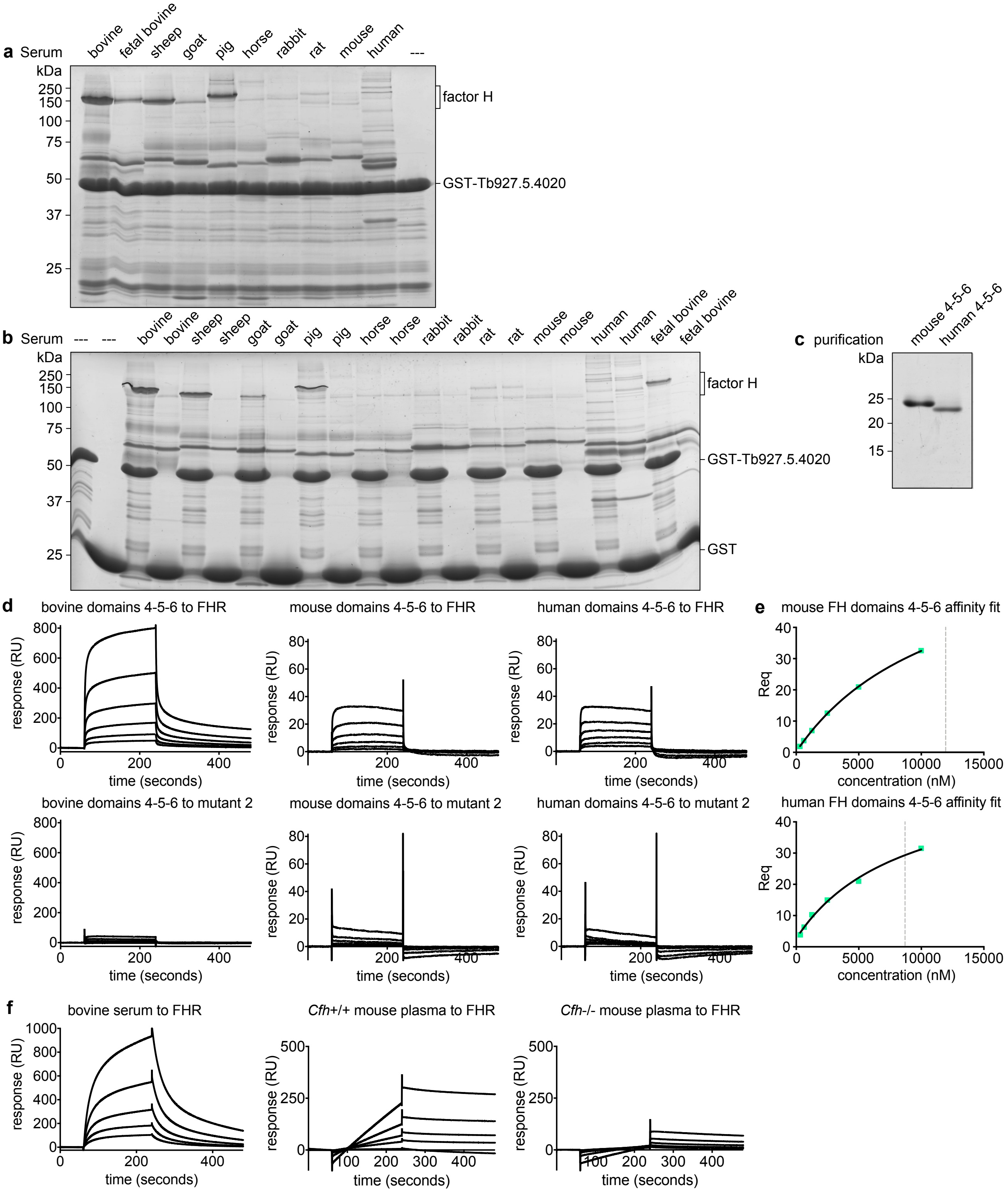

Supplementary Fig. 4 FHR interacts with a range of mammalian FH. a-b SDS-PAGE analysis of pulldowns from a range of mammalian sera were performed with **a** GST-FHR immobilised on beads or **b** along with a GST control. The same GST-FHR samples are run in **a** and **b** and are representative of two independent replicates. **c** SDS-PAGE analysis of recombinant mouse and human FH domains 4-5-6. **d** SPR binding data for C-terminally biotinylated WT FHR (900 RU) and mutant 2 FHR (900 RU) bound to a streptavidin chip and two-fold dilutions of bovine, mouse, and human FH domains 4-5-6 (10 µM highest concentration). **e** Steady state analysis (Req) versus increasing concentrations of mouse and human FH domains 4-5-6 in **d** to determine the K_D (12 µM for mouse and 8.7 µM for human). **f** SPR binding data for C-terminally biotinylated WT FHR (500 RU) bound to a streptavidin chip and 1:10, 1:20, 1:40, 1:80, and 1:160 dilutions of bovine serum and *Cfh*$^{+/+}$ and *Cfh*$^{-/-}$ mouse plasma. Source data are provided as a Source Data file.

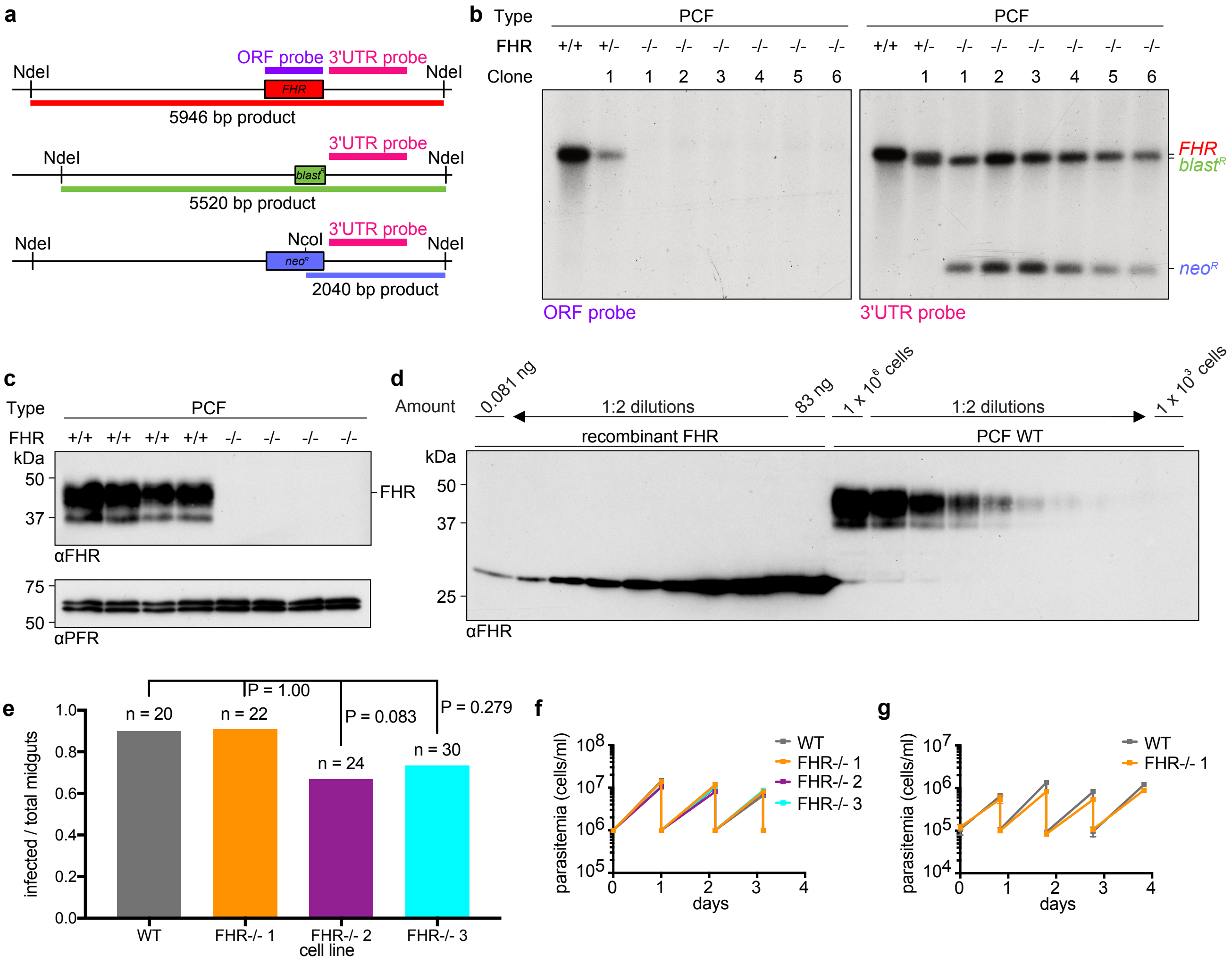

Supplementary Fig. 5 Generation and investigation of FHR-/- cell lines. a Schematic of the FHR locus before and after gene replacement with resistance cassettes showing the NdeI and NcoI restriction sites. **b** Southern blot analysis of genomic DNA from parental and FHR-/- cell lines used to confirm the successful production of six FHR-/- clones. **c** Western blot analysis of FHR expression in four biological replicates using whole cell lysates from cultured PCFs and loading 1 x 10^6 cell equivalents per lane. **d** Estimation of FHR copy number in PCFs using semi-quantitative western blotting. The anti-FHR signal from 1 x 10^6 cell equivalents was roughly equal to 83 ng of recombinant FHR. The limit of detection in this experiment for FHR was 0.081 ng. **c** and **d** were probed with FHR antiserum (αFHR) and anti-PFR (αPFR) as loading control. **e** The transmission of *T. brucei* FHR+/+ and FHR-/- cell lines to tsetse flies in the absence of complement was not significantly different. Tsetse flies were fed FHR+/+ and three independent FHR-/- clones derived as PCFs from culture in the absence of active complement and transmission of the infection was evaluated by the dissection of ≥ 20 midguts per cell line on day 6 post infection. The number of infected midguts over the total dissected midguts is plotted, whereby 18/20 were infected for FHR+/+, 20/22 for FHR-/- clone 1, 16/24 for FHR-/- clone 2, and 22/30 for FHR-/- clone 3. There was no significant difference between the FHR+/+ and each of the FHR-/- clones (P > 0.08 for each cell line by chi-squared test). **f** Growth of FHR+/+ and FHR-/- PCF cell lines in culture. **g** Growth of FHR+/+ and FHR-/- BSF cell lines in culture. Source data are provided as a Source Data file.

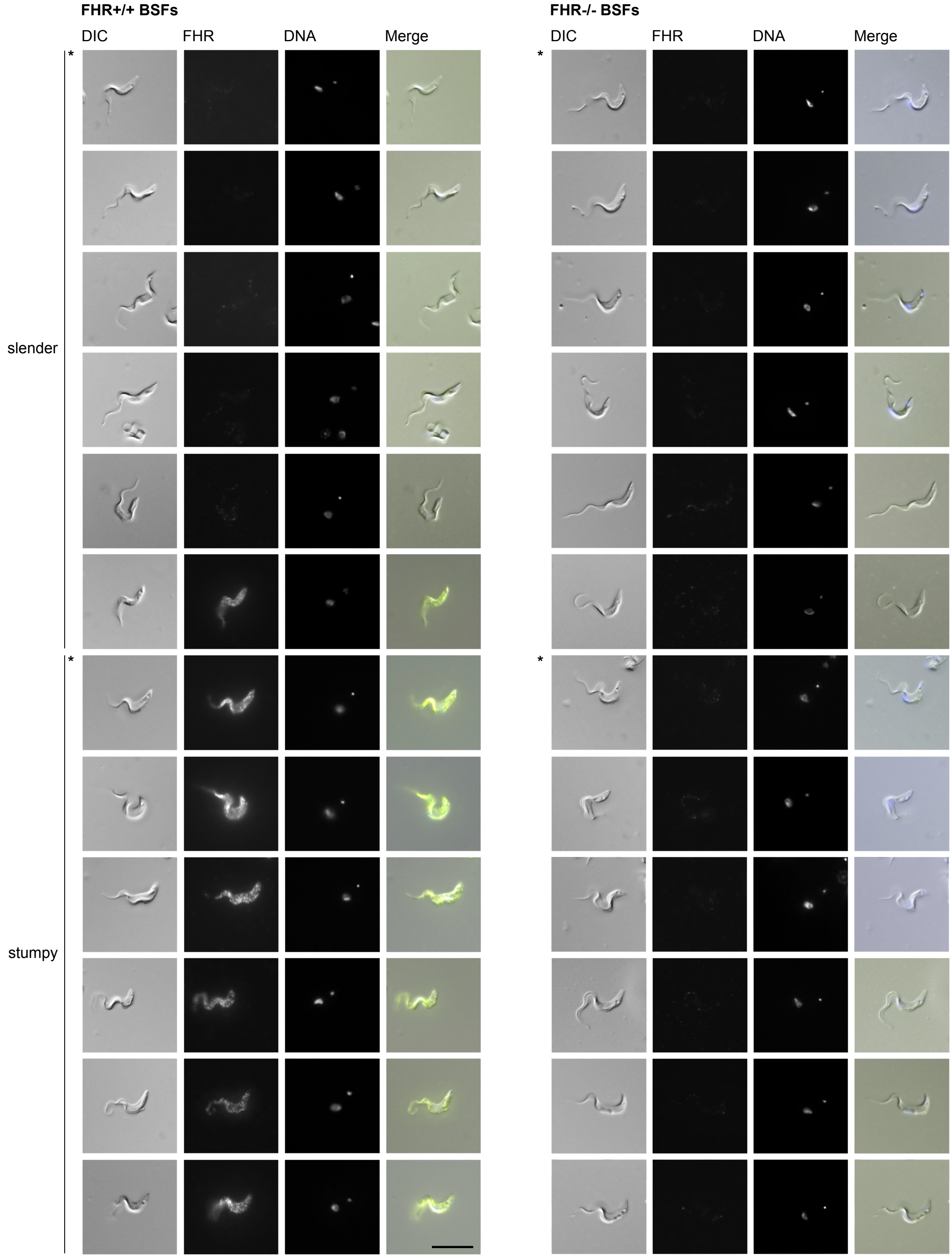

Supplementary Fig. 6 FHR is upregulated and present on the cell surface of induced stumpy BSFs. Immunofluorescence detection of the FHR in FHR+/+ induced stumpy BSFs showing a larger selection of cells than Fig. 5d and representative of a population in which 100% of cells were positive (n=100). FHR expression was not detected in 98% of FHR+/+ slender BSF (n=100). No fluorescence was detected for FHR-/- cells. Cells shown in Fig. 5d are marked by an asterisk (*). Scale bar, 10 µm. DNA, Hoechst staining of the nuclear and kinetoplastid DNA. Cells positioned with the posterior towards the top right corner.

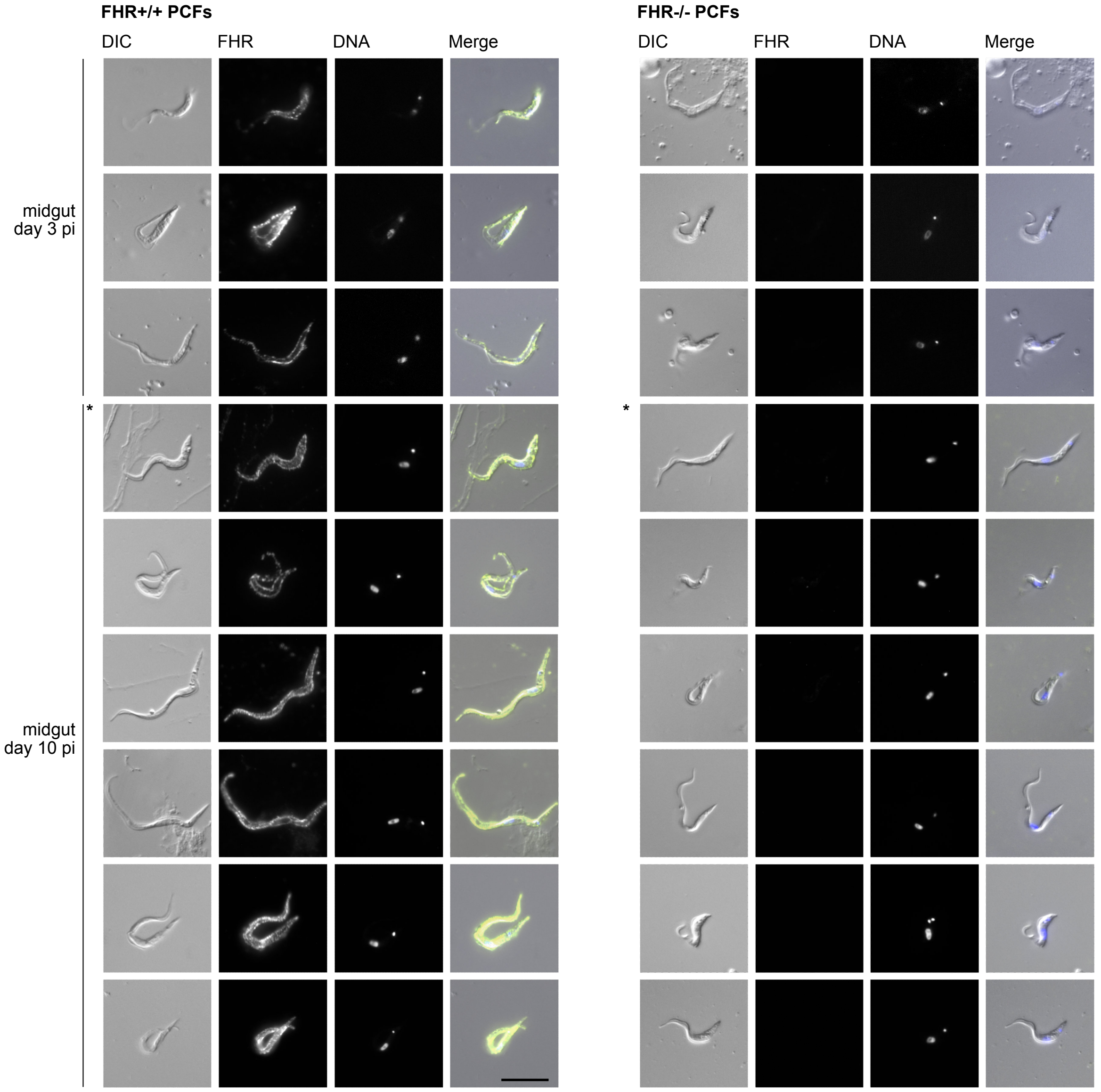

Supplementary Fig. 7 The FHR is upregulated and present on the cell surface of PCFs in the tsetse fly. Immunofluorescence detection of the FHR in FHR+/+ PCFs isolated from the tsetse fly midgut showing a larger selection of cells than Fig. 5d and representative of a population in which 100 % of cells were positive (n = 100 cells). Dissections were performed on days 3 and 10 post infection. No fluorescence was detected for FHR-/- cells. Cells shown in Fig. 5d are marked by an asterisk (*). Scale bar, 10 µm. DNA, Hoechst staining of the nuclear and kinetoplastid DNA. Cells positioned with the posterior towards the top right corner.

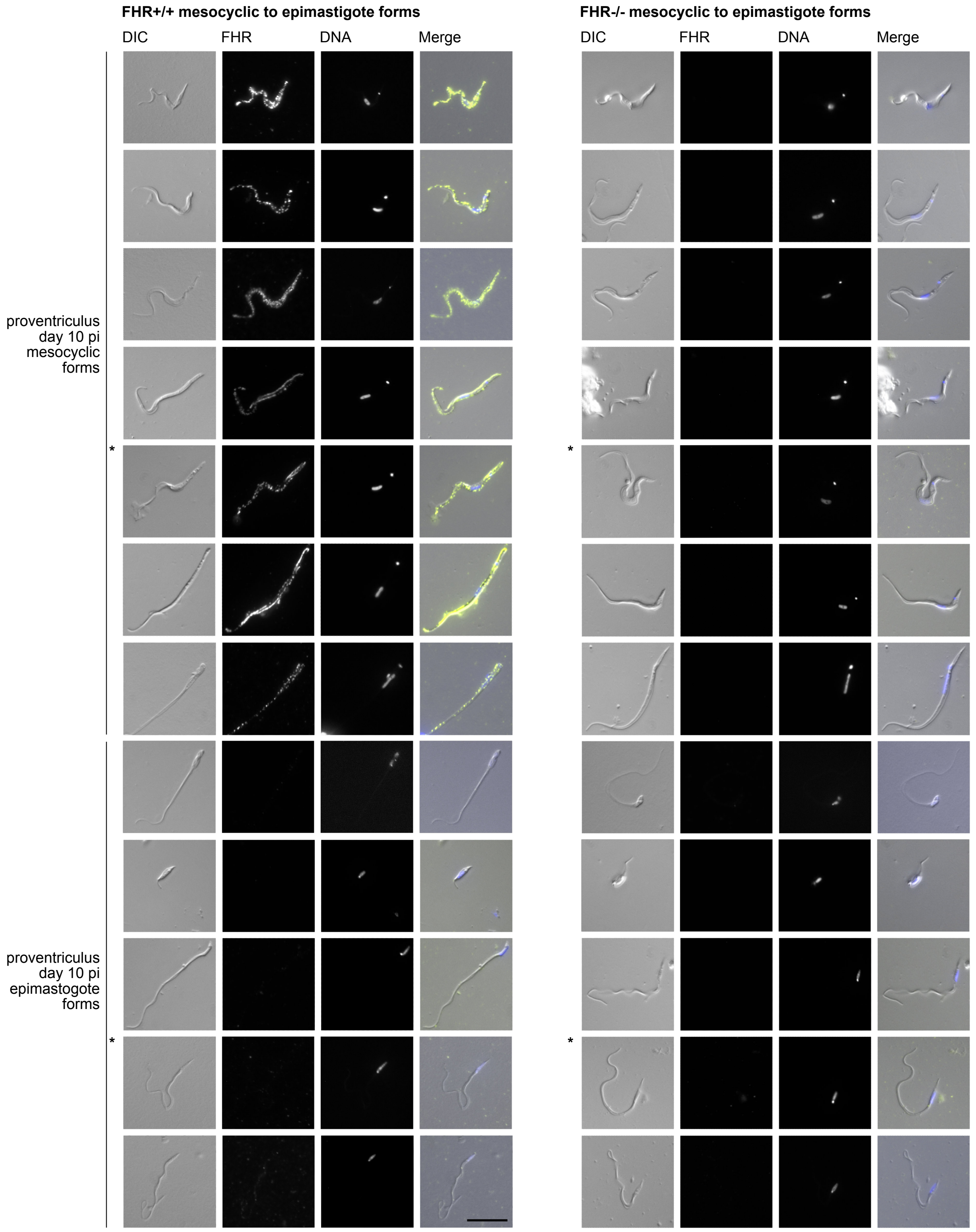

Supplementary Fig. 8 The FHR is upregulated and present on the cell surface of mesocyclic forms, but not detected in epimastigote forms in the tsetse fly. Immunofluorescence detection of the FHR in FHR+/+ mesocyclic forms and epimastigote forms isolated from the tsetse fly proventriculus showing a larger selection of cells than Fig. 5d. Dissections were performed on day 10 post infection. Mesocyclic and epimastigote forms were identified based on the relative positioning of the kinetoplast and nucleus. No fluorescence was detected for FHR-/- cells. Cells shown in Fig. 5d are marked by an asterisk (*). Scale bar, 10 µm. DNA, Hoechst staining of the nuclear and kinetoplastid DNA. Cells positioned with the posterior towards the top right corner.

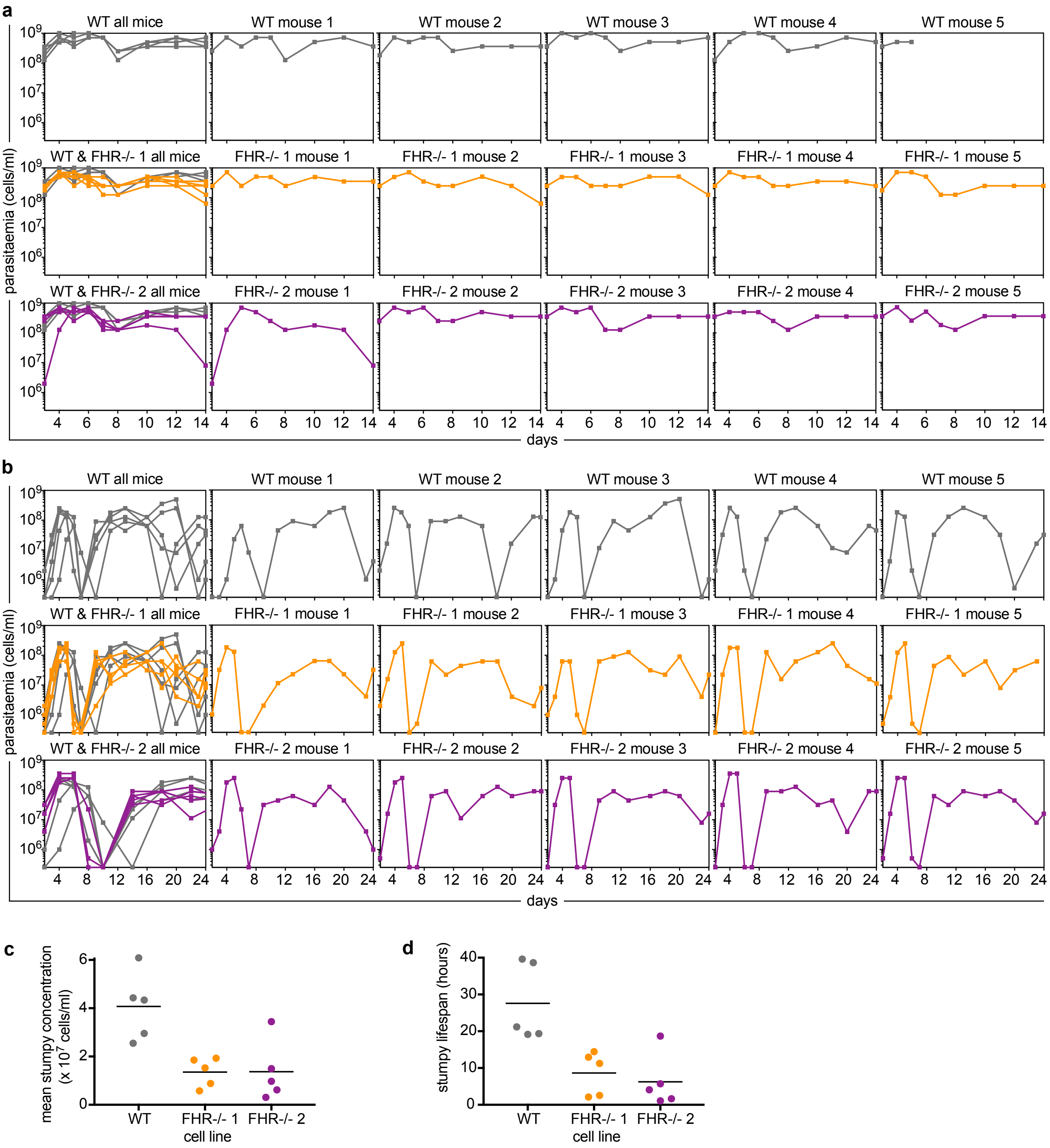

Supplementary Fig. 9 Parasitaemia of *T. brucei* FHR+/+ and two FHR-/- cell lines in mice and predicted stumpy BSF concentration and lifespan. a Mouse blood parasitaemia of parental FHR+/+ and two independent FHR-/- clones from 3 to 14 days post infection. Five immunosuppressed BALB/c mice were infected with each cell line. The parasitaemia for each individual mouse is shown as well as an overlay. **b** Mouse blood parasitaemia of parental FHR+/+ and two independent FHR-/- clones from 2 to 24 days post infection. Five immunocompetent BALB/c mice were infected with each cell line. The parasitaemia for each individual mouse is shown as well as an overlay. **c** Predicted mean stumpy BSF concentration for each individual immunocompetent mouse in **b** from mathematical modelling (Supplementary Fig. 10 and Supplementary Data 1). Horizontal line represents the overall mean value for each cell line. **d** Predicted lifespan of stumpy BSFs in each individual immunocompetent mouse in **b** from mathematical modelling (Supplementary Fig. 10 and Supplementary Data 1). Horizontal line represents the mean value for each cell line. Source data are provided as a Source Data file.

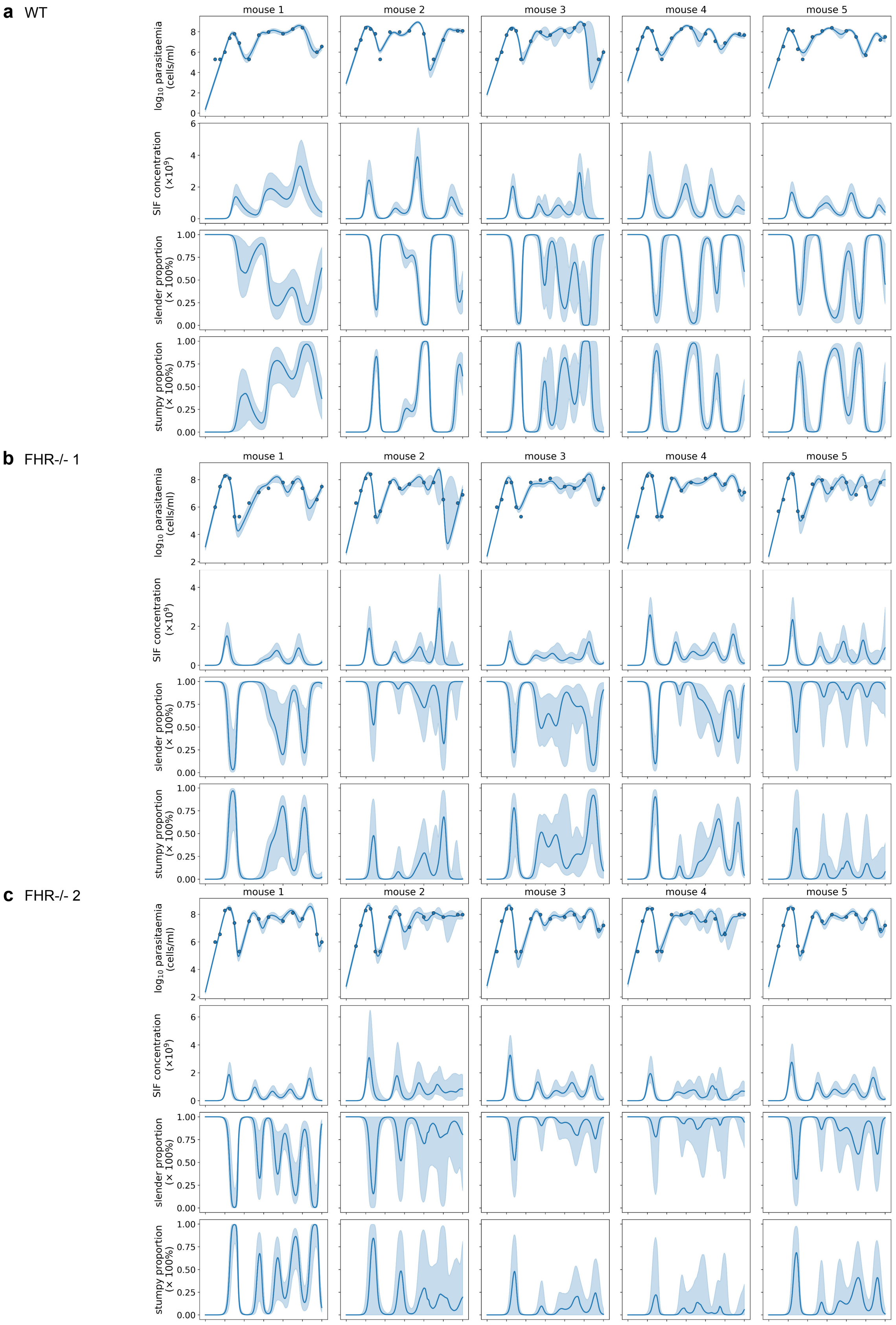

Supplementary Fig. 10 Model dynamics of parasitaemia in immunocompetent mice. Model dynamics are shown for: **a** FHR+/+, **b** FHR-/- clone 1, and **c** FHR-/- clone 2. For all, blue lines show median fits and blue regions show 95% confidence interval. Row 1 shows the model fit to the parasitaemia of individual mice (Supplementary Fig. 9b) projected from days 0 to 24 post infection and blue circles show data. Row 2 shows the predicted stumpy induction factor concentration[1]. Rows 3 and 4 show the predicted proportion of slender and stumpy BSFs. Source data are provided as a Source Data file and for an expanded description of the model see Supplementary Data 1.

Supplementary Table 1 *T. brucei* genome screen for GPI-anchored surface receptors.

GeneID[a]	Invariant surface protein[b]	Actual / structure prediction[c]	Cell surface proteome[d]
Tb927.5.4010	Tb927.5.4010		ESP4
Tb927.5.4020	Tb927.5.4020	3HB	Fam19 & 50
Tb927.6.440	TbbHpHbR	3HB	
Tb927.7.360	Tb927.7.360	3HB	Fam50
Tb927.7.380	Tb927.7.380		
Tb927.7.400	Tb927.7.400		
Tb927.7.420	Tb927.7.420		
Tb927.7.440	Tb927.7.440		
Tb927.8.930	Tb927.8.930	3HB-3HB	Fam50
Tb927.8.950	Tb927.8.950		
Tb927.8.7310	Tb927.8.7310		
Tb927.8.7330	Tb927.8.7330		
Tb927.9.7850	Tb927.9.7850	3HB	Fam51
Tb927.9.7870	Tb927.9.7870		
Tb927.9.7900	Tb927.9.7900		
Tb927.9.7930	Tb927.9.7930		
Tb927.9.7950	Tb927.9.7950		
Tb927.9.13200	Tb927.9.13200		
Tb927.9.15650	Tb927.9.15650	3HB	
Tb927.10.4380	Tb927.10.4380		
Tb927.10.4390	Tb927.10.4390		
Tb927.10.5700	Tb927.10.5700		Fam7 & ESP3
Tb927.10.5710	Tb927.10.5710		
Tb927.11.4070	Tb927.11.4070		
Tb927.11.4760	Tb927.11.4760	3HB	Fam50

[a]Gene ID's have been taken from the TriTryp database[2]. Some genes are single copy genes, while others are members of small gene families.
[b]Protein names are denoted as their gene ID. *Tb927.6.440* encodes the *T. b. brucei* haptoglobin haemoglobin receptor (TbbHpHbR)[3].
[c]Three helical bundle (3HB) indicates predicted structural homology to known trypanosome 3HB surface proteins. The structure for the TbbHpHbR has been determined (PDB 4X0J) and is denoted as 3HB[4]. Mature polypeptides were used to search for structural homologues[5,6].
[d]Data from previous cell surface proteome studies is shown: cell surface phylome family (Fam#)[7] or enriched surface-labelled protein (ESP)[8].

Supplementary Table 2 X-ray data collection and refinement statistics.

**Data collection**	
Space group	C 1 2 1
Cell dimensions	
a, b, c (Å)	160.50, 66.06, 71.02
α, β, γ (°)	90.00, 94.44, 90.00
Resolution (Å)	27.77 (2.70)
R_{merge}	0.157 (0.609)
R_{pim}	0.112 (0.415)
I/ σ(I)	7.4 (2.9)
CC1/2	0.978 (0.740)
Completeness (%)	98.8 (98.5)
Redundancy	3.3 (3.3)
**Refinement**	
Resolution (Å)	2.70
Number of unique reflections	20315 (1049)
R_{work} / R_{free}	0.188 / 0.252
Number of residues	
Protein	544
Water	188
R.m.s. deviations	
Bond lengths (Å)	0.010
Bond angles (°)	1.14
Ramachandran plot	
Favoured (%)	99.3
Allowed (%)	0.7
Disallowed (%)	0

*Values in parentheses are for highest-resolution shell.

Supplementary Table 3 FHR and bovine FH domain 5 interactions in the complex.

Receptor residue	Group	Water link?	Ligand residue	Group	Interaction
N27	side chain OD1 & ND2	yes	S282	side chain OG	hydrogen bond
	side chain OD1 & ND2	yes	K283	main chain N	hydrogen bond
	side chain OD1	yes	K283	main chain O	hydrogen bond
E31	side chain OE1	no	R278	side chain NH1/2	salt bridge
Q34	side chain OE1	no	E280	main chain N	hydrogen bond
S131	side chain OG	no	K296	sidechain NZ	hydrogen bond
E132	main chain O	yes	K296	main chain N	hydrogen bond
V136	side chain	no	V276	side chain	hydrophobic
L140	side chain	no	V276	side chain	hydrophobic
R142	side chain NH1	yes	E293	side chain OE2	hydrogen bond
H147	side chain NE1/2	no	D306	side chain OD1/2	salt bridge
Y184	side chain OH	yes	R278	side chain NH2	hydrogen bond
M185	main chain N	no	E280	side chain OE1/2	hydrogen bond
Q186	side chain NE2	no	E280	side chain OE2	hydrogen bond
	side chain OE1	no	Y277	main chain N	hydrogen bond
F188	main chain N	yes	G275	main chain O	hydrogen bond
	side chain	no	V276	side chain	hydrophobic
E191	side chain OE1/2	no	K295	side chain NZ	salt bridge

Supplementary Table 4 Conservation of domain 5 binding residues in other mammals.										
**Bovine FH domain 5 interactions with FHR[a]**			**Possible conservation of interactions in other mammals[b]**							
**Ligand residue**	**Group**	**Interaction**	**Sheep**	**Goat**	**Pig**	**Horse**	**Rabbit**	**Rat**	**Mouse**	**Human**
S282	side chain OG	hydrogen bond	✓	✓	*	*				
K283	main chain N	hydrogen bond	✓	✓	*	✓	*	✓	*	✓
K283	main chain O	hydrogen bond	✓	✓	*	✓	*	✓	*	✓
R278	side chain NH1/2	salt bridge	✓	✓						
E280	main chain N	hydrogen bond	✓	✓	✓	*	*	*	*	*
K296	sidechain NZ	hydrogen bond	✓	✓	*	*	*	*		*
K296	main chain N	hydrogen bond	✓	✓	*	*	*	*	*	*
V276	side chain	hydrophobic	✓	✓	*	✓		*	*	
V276	side chain	hydrophobic	✓	✓	*	✓		*	*	
E293	side chain OE2	hydrogen bond	✓			✓	✓	✓	✓	*
D306	side chain OD1/2	salt bridge	*	*						
R278	side chain NH2	hydrogen bond	✓	✓	*	*		*	*	*
E280	side chain OE1/2	hydrogen bond	✓	✓	✓					
E280	side chain OE2	hydrogen bond	✓	✓	✓					
Y277	main chain N	hydrogen bond	✓	✓	✓	✓	✓	✓	✓	✓
G275	main chain O	hydrogen bond	✓	✓	✓	✓	✓	✓	✓	✓
V276	side chain	hydrophobic	✓	✓	*	✓		*	*	
K295	side chain NZ	salt bridge	✓	✓	✓		✓	✓		*
		✓	17	16	6	8	4	6	3	4
		*	1	1	9	5	5	7	8	6
		total	18	17	15	13	9	13	11	10

[a]Focusing only on ligand residues from Supplementary Table 3, there are a total of 18 contacts/interactions that bovine FH domain 5 makes with FHR which is mediated by 11 domain 5 residues.

[b]Some bovine residue interactions were absolutely conserved in other mammals (✓), while some residues were not conserved but were replaced by a residue that could perform the same interaction (*). Residues were deemed (*) if the other mammal contained a residue with a side chain that could perform the same interaction or if the interaction involved a main chain nitrogen or oxygen which is independent of side chain. Blank cells represent non-conserved interactions.

Alignment of FH domain 5 from several mammals

```
                 265       275       285       295       305       315
bovine          EITCDPPRIPNGVYRPELSKYRGQDKITYECKKGFFPEIRGTDATCTRDGWVPVPRCAWK
sheep           EIICDPPRIPNGVYRPELSKYRGQDKITYECKKGFIPEIRGTEATCTRDGWAPAPRCAWK
goat            EIICDPPRIPNGVYRPELSKYRGQDKITYKCKKGFIPEIRGTEATCTRDGWAPAPRCAWK
pig             EITCDPPHIPNGFYTPESNRYRTGDRITYHCKEGFYPEIQGNVARCTGNHWSPAPRCTLK
horse           EVTCDPPYIPNGVYSPRRTKHRTEDEIRYECTNGFYPATRGNTARCTSSGWVPSPRCSLK
rabbit          EVTCNAPYIPNGSYLPKRIQHRTGDEIKYECKTGFYPATRGNTARCTGSGWVPGPRCSLK
rat             EMTCLTPYIPNGIYTPHRIKHRIDDEIRYECKNGFYPATRSPVSKCTITGWIPAPRCSLK
mouse           EKRCSPPYILNGIYTPHRIIHRSDDEIRYECNYGFYPVTGSTVSKCTPTGWIPVPRCTLK
human           EKSCDNPYIPNGDYSPLRIKHRTGDEITYQCRNGFYPATRGNTAKCTSTGWIPAPRCTLK
                *  *  * * ** * *    :*  *.* *.*  ** *   .  : **   * * ***: *
```

Supplementary references

1. Rojas, F. *et al.* Oligopeptide signaling through TbGPR89 drives trypanosome quorum sensing. *Cell* **176**, 306–317 (2019).
2. Aslett, M. *et al.* TriTrypDB: a functional genomic resource for the Trypanosomatidae. *Nucleic Acids Res.* **38**, D457-462 (2010).
3. Vanhollebeke, B. *et al.* A haptoglobin-hemoglobin receptor conveys innate immunity to Trypanosoma brucei in humans. *Science* **320**, 677–681 (2008).
4. Lane-Serff, H., MacGregor, P., Lowe, E. D., Carrington, M. & Higgins, M. K. Structural basis for ligand and innate immunity factor uptake by the trypanosome haptoglobin-haemoglobin receptor. *Elife* **3**, e05553 (2014).
5. Shi, J., Blundell, T. L. & Mizuguchi, K. FUGUE: Sequence-structure homology recognition using environment-specific substitution tables and structure-dependent gap penalties. *J. Mol. Biol.* **310**, 243–257 (2001).
6. Kelley, L. A., Mezulis, S., Yates, C. M., Wass, M. N. & Sternberg, M. J. E. The Phyre2 web portal for protein modeling, prediction and analysis. *Nat. Protoc.* **10**, 845–858 (2015).
7. Jackson, A. P. *et al.* A cell-surface phylome for African trypanosomes. *PLoS Negl. Trop. Dis.* **7**, e2121 (2013).
8. Gadelha, C. *et al.* Architecture of a host-parasite interface: complex targeting mechanisms revealed through proteomics. *Mol. Cell. Proteomics* **14**, 1911–1926 (2015).
