# Supplementary material for: A receptor for the complement regulator factor H increases transmission of trypanosomes to tsetse flies
Source: Nat Commun. 2020 Mar 12;11:1326. doi: 10.1038/s41467-020-15125-y (PMC7067766; doi:10.1038/s41467-020-15125-y)
Supplement: Supplementary file 3 — Reporting Summary [file 41467_2020_15125_MOESM3_ESM.pdf]

nature research

Corresponding author(s): Mark Carrington
Matthew K. Higgins

Last updated by author(s): Jan 31, 2020

Reporting Summary

Nature Research wishes to improve the reproducibility of the work that we publish. This form provides structure for consistency and transparency in reporting. For further information on Nature Research policies, see Authors & Referees and the Editorial Policy Checklist .

Statistics

For all statistical analyses, confirm that the following items are present in the figure legend, table legend, main text, or Methods section.

n/a	Confirmed	
☐	☒	The exact sample size (n) for each experimental group/condition, given as a discrete number and unit of measurement
☐	☒	A statement on whether measurements were taken from distinct samples or whether the same sample was measured repeatedly
☐	☒	The statistical test(s) used AND whether they are one- or two-sided<br>*Only common tests should be described solely by name; describe more complex techniques in the Methods section.*
☒	☐	A description of all covariates tested
☒	☐	A description of any assumptions or corrections, such as tests of normality and adjustment for multiple comparisons
☐	☒	A full description of the statistical parameters including central tendency (e.g. means) or other basic estimates (e.g. regression coefficient) AND variation (e.g. standard deviation) or associated estimates of uncertainty (e.g. confidence intervals)
☐	☒	For null hypothesis testing, the test statistic (e.g. F, t, r) with confidence intervals, effect sizes, degrees of freedom and P value noted<br>*Give P values as exact values whenever suitable.*
☐	☒	For Bayesian analysis, information on the choice of priors and Markov chain Monte Carlo settings
☒	☐	For hierarchical and complex designs, identification of the appropriate level for tests and full reporting of outcomes
☒	☐	Estimates of effect sizes (e.g. Cohen's d, Pearson's r), indicating how they were calculated

Our web collection on statistics for biologists contains articles on many of the points above.

Software and code

Policy information about availability of computer code

Data collection	Data collection and processing software used are described in the methods section and are commercially available or openly accessible.
Data analysis	Data analysis was performed as described in the methods section using commercially available or openly accessible software and code (see Supplementary Data 1). GraphPad Prism version 7 was used to generate graphs, PyMOL for crystallographic structures, and Affinity Photo and Designer to generate figures.

For manuscripts utilizing custom algorithms or software that are central to the research but not yet described in published literature, software must be made available to editors/reviewers. We strongly encourage code deposition in a community repository (e.g. GitHub). See the Nature Research guidelines for submitting code & software for further information.

Data

Policy information about availability of data

All manuscripts must include a data availability statement. This statement should provide the following information, where applicable:

- Accession codes, unique identifiers, or web links for publicly available datasets
- A list of figures that have associated raw data
- A description of any restrictions on data availability

Crystallographic data has been deposited into the Protein Data Bank (PDB) with accession number 6XZ6. The code in this work is deposited on GitHub and details can be found in Supplementary Data 1. All underlying source and raw data can be found in the Source Data file. All other data are available from the authors at request.

Field-specific reporting

Please select the one below that is the best fit for your research. If you are not sure, read the appropriate sections before making your selection.

- [x] Life sciences
- [ ] Behavioural & social sciences
- [ ] Ecological, evolutionary & environmental sciences

For a reference copy of the document with all sections, see nature.com/documents/nr-reporting-summary-flat.pdf

Life sciences study design

All studies must disclose on these points even when the disclosure is negative.

Sample size	Sample sizes for animal and insect model experiments were chosen to yield statistically significant results and are stated in the figure legends or methods section.
Data exclusions	No data were excluded from the analysis.
Replication	Experimental findings were repeated when stated in the figure legends or methods section. All repeats were successful and described in this work.
Randomization	Organisms were sorted randomly and independently prior to allocation to and investigation within experimental groups.
Blinding	Investigators were blinded to the samples when counting and assessing parasitaemia.

Reporting for specific materials, systems and methods

We require information from authors about some types of materials, experimental systems and methods used in many studies. Here, indicate whether each material, system or method listed is relevant to your study. If you are not sure if a list item applies to your research, read the appropriate section before selecting a response.

Materials & experimental systems

n/a	Involved in the study	
[ ]	[x]	Antibodies
[ ]	[x]	Eukaryotic cell lines
[x]	[ ]	Palaeontology
[ ]	[x]	Animals and other organisms
[x]	[ ]	Human research participants
[x]	[ ]	Clinical data

Methods

n/a	Involved in the study	
[x]	[ ]	ChIP-seq
[x]	[ ]	Flow cytometry
[x]	[ ]	MRI-based neuroimaging

Antibodies

Antibodies used	The factor H receptor monoclonal antibody and antiserum was generated as described in the methods section. The paraflagellar rod loading control was a kind gift of Keith Gull (Kohl, L., Sherwin, T. & Gull, K. J. Eukaryot. Microbiol. 46, 105–109 (1999)). All secondary antibodies are commercially available from Jackson Laboratories.
Validation	All antibodies were validated within this work using the conditions outlined.

Eukaryotic cell lines

Policy information about cell lines

Cell line source(s)	Trypanosoma brucei brucei strain J10 was provided by Wendy Gibson (Gibson, W. C., De, T. F. & Godfrey, D. G. Adv. Parasitol. 18, 175–246 (1980)). All other cell lines were analysed as cell lysates and provided as described in this work.
Authentication	Results independent of isolate identity.
Mycoplasma contamination	Mycoplasma does not contaminate trypanosome cultures.
Commonly misidentified lines (See ICLAC register)	No commonly misidentified cell lines were used in this work.

Animals and other organisms

Policy information about studies involving animals; ARRIVE guidelines recommended for reporting animal research

Laboratory animals	Age matched BALB/c female mice were used. Female and male Glossina morsitans morsitans and Glossina pallidipes, and female Glossina palpalis gambiensis were used as described.
Wild animals	The study did not involve wild animals.
Field-collected samples	The study did not involve samples collected from the field.
Ethics oversight	All animal and insect investigations were performed in accordance with and authorised by the University of Bristol's Ethics of Research Committee (PPL 30/3046) and the regional Ethic Committee for Animal Experimentation (CEEA-LR 36, Montpellier, PPL 2018012915201897v2). All experiments were in accordance with the UK Animals (Scientific) Procedures act and the French Ministry for Higher Education and Research.

Note that full information on the approval of the study protocol must also be provided in the manuscript.
