# Supplementary material for: A receptor for the complement regulator factor H increases transmission of trypanosomes to tsetse flies
Source: Nat Commun. 2020 Mar 12;11:1326. doi: 10.1038/s41467-020-15125-y (PMC7067766; doi:10.1038/s41467-020-15125-y)
Supplement: Supplementary file 4 — Description of Additional Supplementary Files [file 41467_2020_15125_MOESM4_ESM.pdf]

Description of Additional Supplementary Files

File Name: Supplementary Data 1
Description: containing a description of the mathematical model used in main text.
