# Supplementary material for: A receptor for the complement regulator factor H increases transmission of trypanosomes to tsetse flies
Source: Nat Commun. 2020 Mar 12;11:1326. doi: 10.1038/s41467-020-15125-y (PMC7067766; doi:10.1038/s41467-020-15125-y)
Supplement: Supplementary file 5 — Supplementary Data 1 [file 41467_2020_15125_MOESM5_ESM.pdf]

Supplementary Material: A receptor for the complement regulator factor H increases transmission of trypanosomes to tsetse flies

Olivia J. S. Macleod, Jean-Mathieu Bart, Paula MacGregor, Lori Peacock, Nicholas J. Savill, Svenja Hester, Sophie Ravel, Jack D. Sunter, Camilla Trevor, Steven Rust, Tristan J. Vaughan, Ralph Minter, Shabaz Mohammed, Wendy Gibson, Martin C. Taylor, Matthew K. Higgins, Mark Carrington

1 A model of antigenic variant dynamics

A schematic of the model is shown in Fig. 1. Slender cells produce SIF which causes their differentiation into stumpy cells via an intermediate, committed slender cell population. Stumpy cells have a natural limited lifespan. Slender cells of antigenic variant 1 induce an immune response which kills all cells of this variant. Slender cells of variant 1 switch at a fixed rate to variant 2, and similarly, slender cells of variant 2 can switch to variant 3 at a different rate. Analysis of the model fits suggests that just three variants are required to give excellent agreement to the data. These variants produce SIF which enters a common pool. We include a variant 2-induced immune response in the model that kill cells of variant 2. But we do not include a variant 3-induced immune response in the model because the time series are too short to provide sufficient information to parametrise this response.

We initialise the model on day 0 for each mouse with only slender cells of variant 1. SIF concentration and all immune responses are set to zero.

The model used in this paper is a simpler version of that used in [3]. Let the concentration of non-committed slender cells of variant type v at time t be $l_v(t)$. The initial infection by variant type 1 is at time $t = 0$. Switching between variants is sequential, i.e., variant v switches to variant $v+1$ and no other.

Non-committed slender cells replicate at rate α (i.e., a cell-cycle time of $\frac{\ln 2}{\alpha}$). They are cleared by a time-dependent, variant-specific immune response at rate $\delta I_v(t)$. They become committed to differentiate at rate $\beta f(t)$, where $f(t)$ is SIF concentration, and β a constant of proportionality. Switching from variant v to variant $v+1$ is at variant specific rate ω_v. Thus the differential equations that describe the dynamics of each non-committed slender variant are

$$\frac{d}{dt}l_1(t) = [\alpha - \beta f(t) - \omega_1 - \delta I_1(t)]l_1(t) \tag{1}$$

$$\frac{d}{dt}l_2(t) = [\alpha - \beta f(t) - \omega_2 - \delta I_2(t)]l_2(t) + \omega_1 l_1(t) \tag{2}$$

$$\frac{d}{dt}l_3(t) = [\alpha - \beta f(t)]l_3(t) + \omega_2 l_2(t) \tag{3}$$

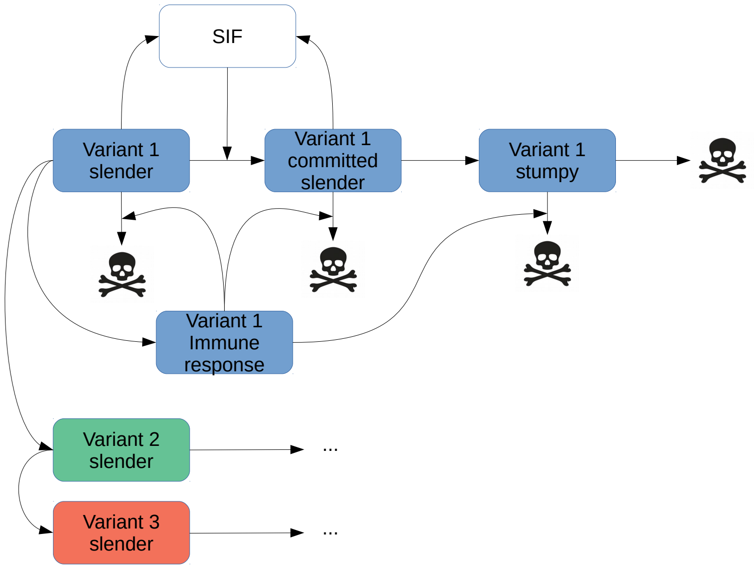

Figure 1: Schematic of the mathematical model.

Let a be the age of differentiated cells since becoming committed to differentiation, and let $d_v(a,t)$ be the age distribution of differentiated cells of variant type v at time t.

Once committed to differentiation, slender cells retain a slender morphology for τ_l hours and can still replicate at rate α. After τ_l hours they differentiate into non-replicating stumpy cells and can survive for a further τ_s hours, at which point they are assumed to die and are removed from the population. All committed cells of variant type v are cleared by the immune system at a rate $I_v(t)$. Thus the partial differential equations that describe the dynamics of the age distribution of each differentiated variant type v are

$$\frac{\partial}{\partial t}d_v(a,t) + \frac{\partial}{\partial a}d_v(a,t) = d_v(a,t) \times \begin{cases} -I_v(t) + \alpha & \text{if } 0 \leq a < \tau_l \\ -I_v(t) & \text{if } \tau_l \leq a < \tau_s \end{cases} \tag{4}$$

The boundary conditions on these equations are determined by differentiation of non-committed slender cells into age $a = 0$, i.e., $d_v(0,t) = \beta f(t) l_v(t)$, and stumpy death at age τ_s, i.e., $d_v(\tau_s, t) = 0$.

Let $L(t)$ be the total concentration of non-committed slender cells. It is given by the sum over all variants

$$L(t) = \sum_{v=1}^{3} l_v(t) \tag{5}$$

Let $D(a,t)$ be the age distribution of differentiated cells of all variant types at

time t. It is given by the sum over all variants

$$D(a,t) = \sum_{v=1}^{3} d_v(a,t) \tag{6}$$

Let $C(t)$ be the total concentration of committed slender cells, let $S(t)$ be the total concentration of stumpy cells, and let $T(t)$ be the total concentration of cells. These are given by

$$C(t) = \int_0^{\tau_l} D(a,t)\, da \tag{7}$$

$$S(t) = \int_{\tau_l}^{\tau_s} D(a,t)\, da \tag{8}$$

$$T(t) = L(t) + C(t) + S(t) \tag{9}$$

SIF is produced by committed and non-committed slender cells of all variants at a rate of 1 unit of SIF per cell per hour. SIF is lost at rate γ. Thus the differential equation describing the dynamics of SIF concentration is

$$\frac{d}{dt} f(t) = L(t) + C(t) - \gamma f(t) \tag{10}$$

The immune response against trypanosomes is multifactorial and highly complex. A detailed mathematical model of the immune response is, therefore, of little use when no data is available to fit to. Instead, we use a single variable $I_v(t)$, that determines the clearance rate of each variant type. We assume that the immune response against variant type v is activated at a rate $\psi l_v(t)$ by replicating slender cells of variant type v. The differential equation that describe the immune-mediated clearance rate of variant type v is therefore

$$\frac{d}{dt} I_v(t) = \psi l_v(t) \tag{11}$$

Naive mice are infected with non-committed slender cells of variant type 1 at a concentration l_0. Therefore the initial conditions are $l_1(0) = l_0$, $l_2(0) = l_3(0) = 0$, $d_v(a,0) = 0$ for all v and a, $I_v(0) = 0$ for all v, and $f(0) = 0$. These imply $L(0) = l_0$, $D(a,0) = 0$ for all a and $C(0) = S(0) = T(0) = 0$.

All variables and parameters are listed in Table 1.

2 Model fits

Parasite concentration was estimated microscopically using the rapid matching method [2] which attempts to roughly estimate the number of organisms in one or more microscope fields. The number of parasites observed in F microscope fields given a predicted concentration L, is Poisson distributed with parameter $32FL \times 10^{-8.1}$. The reference value $32 \times 10^{-8.1}$, is given in [2]. The model is fitted to each mouse using an adaptive population based Markov chain Monte Carlo algorithm with power posteriors [1]. The computer code for the model is available at https://github.com/nicksavill/An-African-trypanosome-receptor-for-factor-H-increases-transmission-to-the-tsetse-fly-vector and the MCMC code is available at https://github.com/nicksavill/bayesian-dynamical-model-inference.

**Variables**		
t	time since infection	h
a	age of differentiated cells	h
**Dependent variables**		
$l_v(t)$	concentration of non-committed slender cells of variant type v	cells ml^{-1}
$d_v(a,t)$	age distribution of differentiated cells of variant type v	cells ml^{-1}h^{-1}
$I_v(t)$	immune-mediated clearance rate of variant type v	h^{-1}
$f(t)$	SIF concentration	dimensionless
$L(t)$	total concentration of non-committed slender cells	cells ml^{-1}
$C(t)$	total concentration of committed slender cells	cells ml^{-1}
$S(t)$	total concentration of stumpy cells	cells ml^{-1}
$T(t)$	total concentration of all cells	cells ml^{-1}
$D(a,t)$	age distribution of differentiated cells	cells ml^{-1}h^{-1}
**Parameters**		
α	replication rate of slender cells	h^{-1}
β	differentiation rate	h^{-1}
γ	SIF removal rate	h^{-1}
δ	proportionality constant for immune-mediated clearance of stumpy cells	dimensionless
τ_l	duration of committed slender cell stage	h
τ_s	duration of stumpy cell stage	h
ψ	committed slender cell-induced growth rate of immune response	ml cell^{-1} h^{-1}
λ	initial concentration of variant type 1	cells ml^{-1}
ω_v	switch rate from variant type v to variant type $v+1$	h^{-1}

Table 1: Variables and parameters used in the model

The fits of the model to each wildtype, FHR-/-1 and FHR-/-2 mouse (columns) are shown in Figs. 2-4. The top panels show the fit of the model to log base 10 parasitaemia (circles: data, line: median fit, coloured area: 95% CI). The second row of panels shows the predicted SIF concentration in arbitrary units. SIF causes differentiation from slender to stumpy forms, and so peaks in SIF correlate to (slightly lagged) drops in slender proportion (third row of panels) and rises in stumpy proportion (fourth row of panels). Of particular interest are the generally higher stumpy peaks in wildtype mice than in FHR-/- mice due to the longer lifespan of stumpy forms in wildtype mice. The fifth and sixth rows of panels show the predicted immune responses of variants 1 and 2. There is insufficient data to predict the variant-3 immune response. Note the step-like rise in immune responses that correspond to successive peaks in variant parasitaemias (rows 7 and 8).

In conclusion, peaks in a variant's parasitaemia arise due to SIF-induced differentiation of slender forms to short-lived stumpy forms and slender form rebound due to fast degradation of SIF. Each variant peak induces a cumulative increase in the immune response against that variant which, over time, causes reduction in peak height. Immune clearance of a variant, coupled with switching to another variant, causes the successive replacement of one variant by another.

3 Parameter estimates

Parameter estimates for each mouse in each cell line are shown in Fig. 5. Apart from stumpy duration, all other parameters show no significant differences between cell lines.

References

[1] Caroline E. Dewar, Paula MacGregor, Sinclair Cooper, Matthew K. Gould, Keith R. Matthews, Nicholas J. Savill, and Achim Schnaufer. Mitochondrial DNA is critical for longevity and metabolism of transmission stage *Trypanosoma brucei*. *PLoS Pathogens*, 14(7), 2018.

[2] W. J. Herbert. *Trypanosoma brucei*: A rapid "matching" method for estimating the host's parasitemia. *Experimental Parasitology*, 40(3):427–431, 1976.

[3] Paula MacGregor, Nicholas J. Savill, Deborah Hall, and Keith R. Matthews. Transmission stages dominate trypanosome within-host dynamics during chronic infections. *Cell Host and Microbe*, 9(4):310–318, 2011.

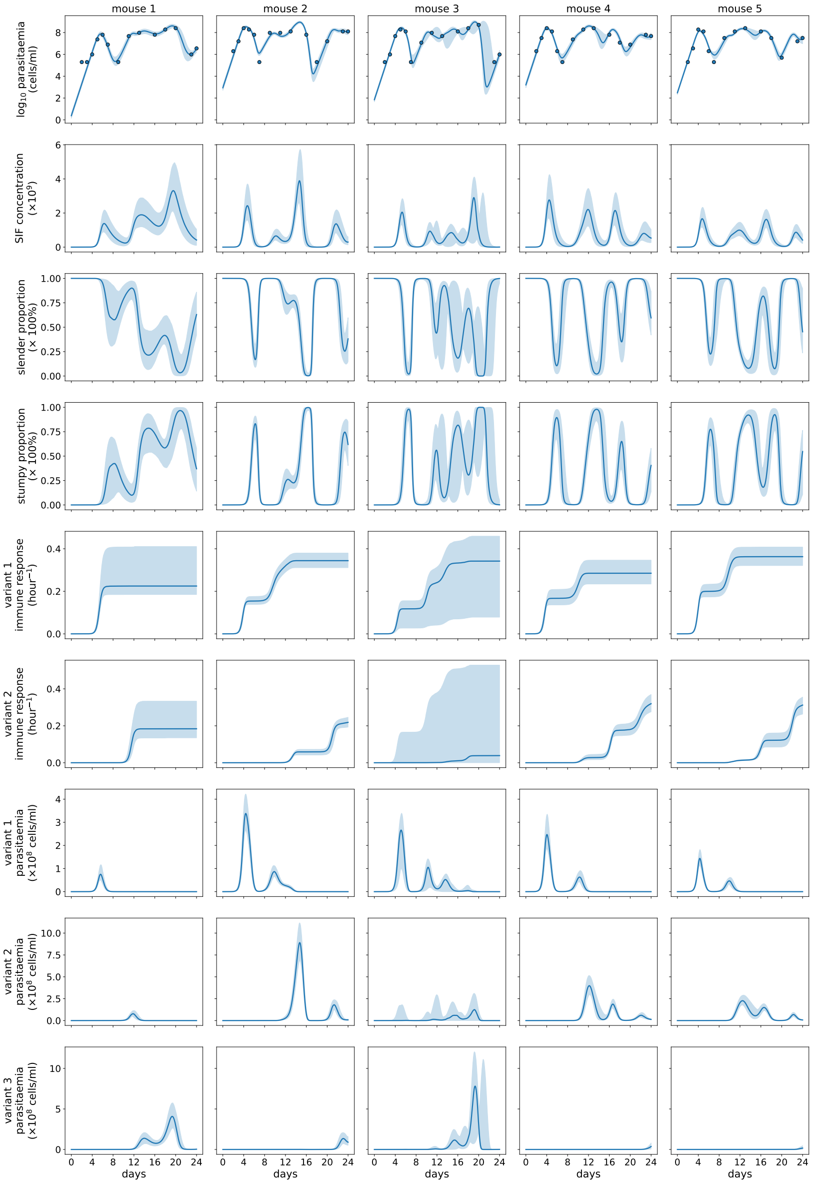

Figure 2: Model dynamics of the wildtype cell line in immunocompetent mice.

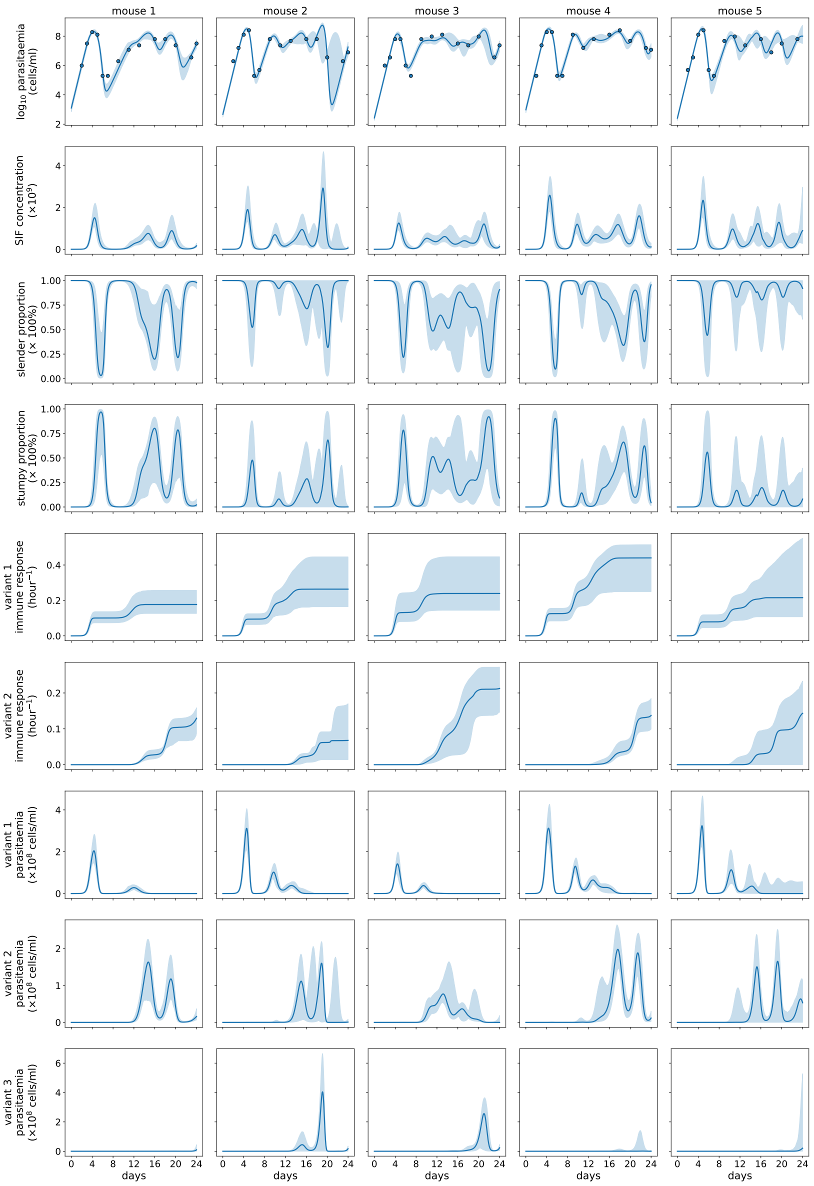

Figure 3: Model dynamics of cell line FHR-/-1 in immunocompetent mice.

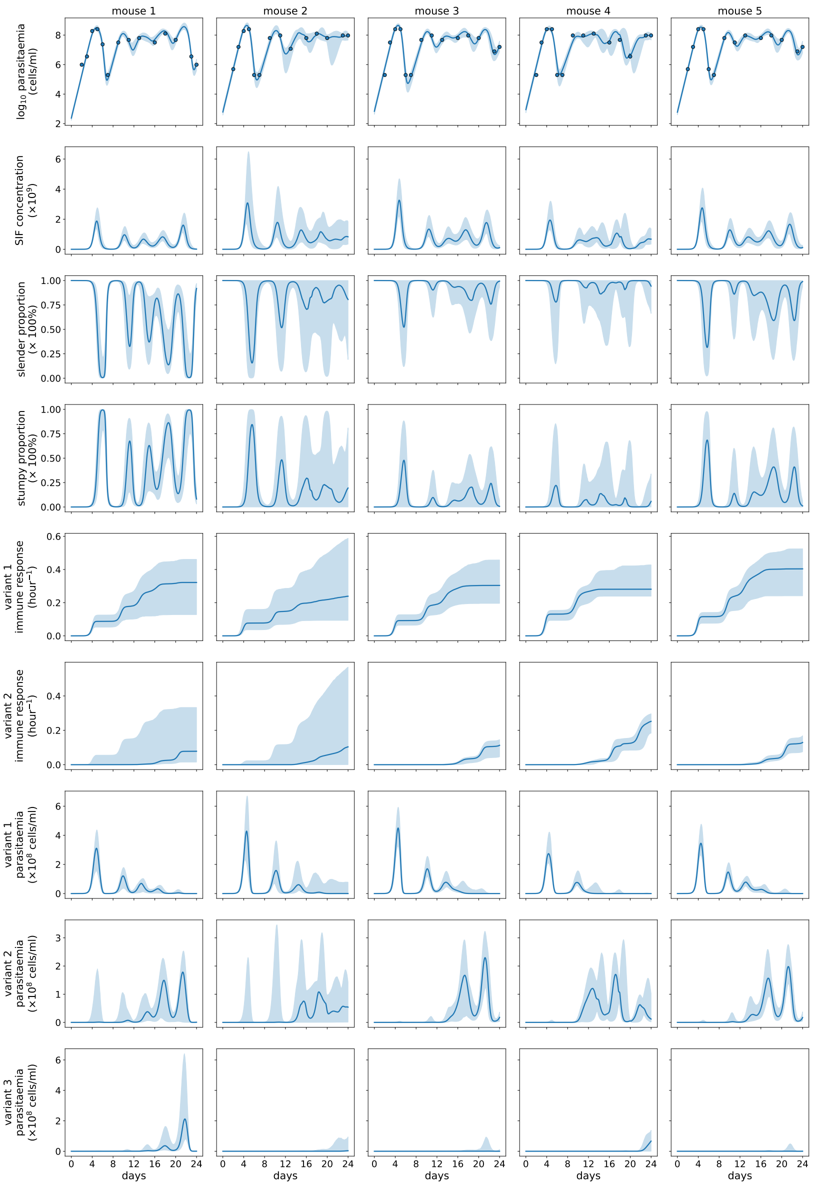

Figure 4: Model dynamics of the cell line FHR-/-2 in immunocompetent mice.

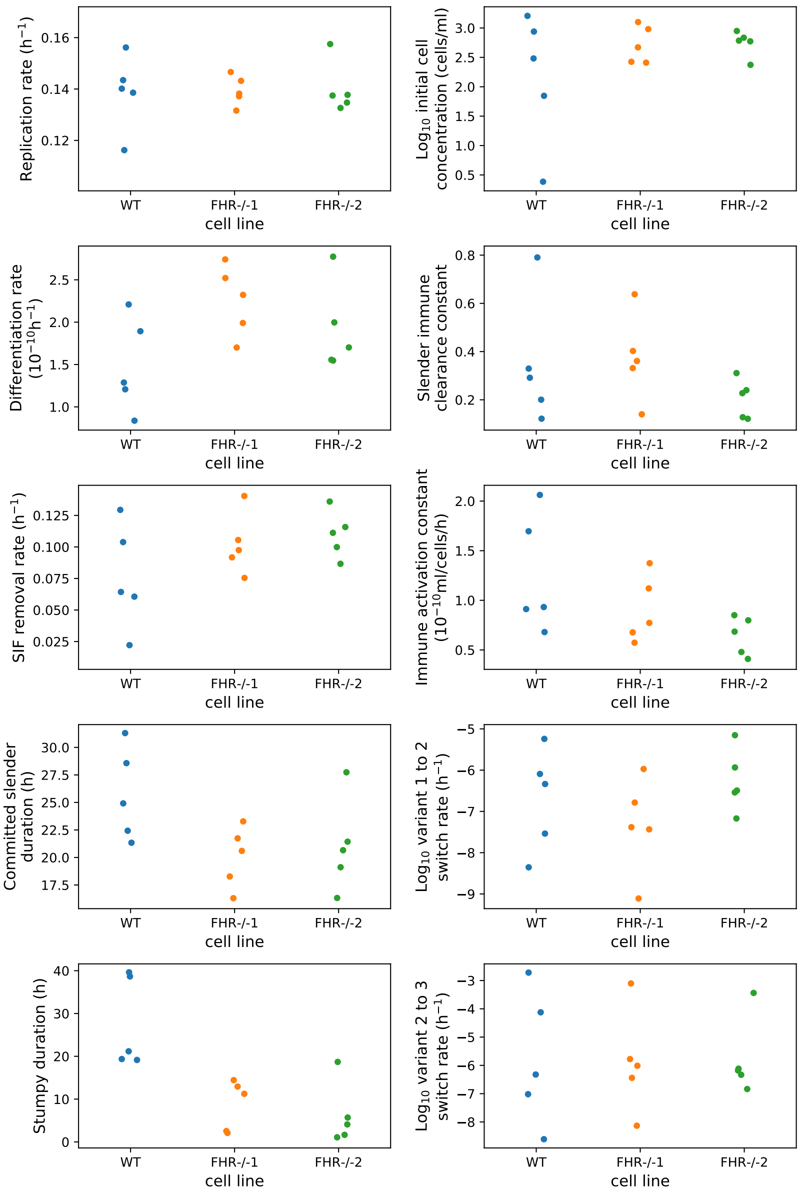

Figure 5: Mean estimated parameter values for each mouse (circles) in each cell line.
